# Evaluation of simplified acute physiology score 3 performance: a systematic review of external validation studies

**DOI:** 10.1186/cc13911

**Published:** 2014-06-06

**Authors:** Antonio Paulo Nassar, Luiz Marcelo Sa Malbouisson, Rui Moreno

**Affiliations:** 1 Anesthesiology Department, Hospital das Clinicas SP-FMUSP, Av. Dr. Enéas de Carvalho Aguiar, 255, Cerqueira César, São Paulo, SP 05403-000, Brazil; 2 Unidade de Cuidados Intensivos Neurocríticos, Hospital de São José, Centro Hospitalar de Lisboa Central, Faculdade de Ciências Médicas de Lisboa, Universidade Nova de Lisboa, EPE, 1169-050 Lisbon, Portugal

## Abstract

**Introduction:**

Simplified Acute Physiology Score 3 (SAPS 3) was the first critical care prognostic model developed from worldwide data. We aimed to systematically review studies that assessed the prognostic performance of SAPS 3 general and customized models for predicting hospital mortality in adult patients admitted to the ICU.

**Methods:**

Medline, Lilacs, Scielo and Google Scholar were searched to identify studies which assessed calibration and discrimination of general and customized SAPS 3 equations. Additionally, we decided to evaluate the correlation between trial size (number of included patients) and the Hosmer-Lemeshow (H-L) statistics value of the SAPS 3 models.

**Results:**

A total of 28 studies were included. Of these, 11 studies (42.8%) did not find statistically significant mis-calibration for the SAPS 3 general equation. There was a positive correlation between number of included patients and higher H-L statistics, that is, a statistically significant mis-calibration of the model (r = 0.747, *P* <0.001). Customized equations for major geographic regions did not have statistically significant departures from perfect calibration in 9 of 19 studies. Five studies (17.9%) developed a regional customization and in all of them this new model was not statistically different from a perfect calibration for their populations. Discrimination was at least very good in 24 studies (85.7%).

**Conclusions:**

Statistically significant departure from perfect calibration for the SAPS 3 general equation was common in validation studies and was correlated with larger studies, as should be expected, since H-L statistics (both C and H) are strongly dependent on sample size This finding was also present when major geographic customized equations were evaluated. Local customizations, on the other hand, improved SAPS 3 calibration. Discrimination was almost always very good or excellent, which gives excellent perspectives for local customization when a precise local estimate is needed.

## Introduction

Prognostic models are important tools in critical care medicine [1]. They are used for mortality predictions and for illness severity assessment in clinical trials. Acute physiology and chronic health evaluation II (APACHE II) [2] and simplified acute physiology score II (SAPS II) [3] are the most commonly used models worldwide. Although they are still able to assess severity in clinical trials, their usefulness for mortality predictions has been questioned due to a lack of prognostic performance over time, since they were developed more than 20 years ago. This limitation is very important because observed-to-expected mortality ratios have become standard to assess the impact of ICU factors on outcome and are among the safety and quality indicators that an ICU should apply to evaluate quality of care [4].

In 2002, a worldwide group of researchers collected new data about physiologic alterations, clinical presentation and outcome of critically ill patients in more than 300 ICUs worldwide [5]. These data led to the development of a new prognostic model, the SAPS III [6]. For the first time, a general outcome prediction model included data from outside Europe or the USA. Besides the general equation for mortality estimation, SAPS III also provided the end-user with customized equations for seven different regions of the world, which would theoretically improve care quality evaluations and benchmarking.

Since then, SAPS III models have been prospectively evaluated for their performance in several regional studies. Our aim was to systematically review studies that assessed the prognostic performance of SAPS III general and customized models for predicting hospital mortality in adult patients admitted to ICU. Specifically, we aimed to review how SAPS III calibrated and discriminated in external validation studies.

## Methods

### Search strategy

Medline, Lilacs (*Literatura Latino-Americana e do Caribe em Ciências da Saúde*) and Scielo (*Scientific Electronic Library Online*) were searched for articles published from 1 January 2005 (because the original SAPS III description was published in that year) to 1 October 2013 using the term ‘simplified acute physiology scoreʼ. Besides that, Google Scholar was searched for articles that cited the original SAPS III publication [6]. Titles and abstracts returned by the search strategy were analyzed for eligibility and full-text copies of articles deemed to be potentially relevant were retrieved. Duplicate publications were excluded, as well as studies published only as abstracts and editorials, letters and narrative reviews. Only articles published in English, Portuguese, Spanish or French were included. Eligibility assessment was performed independently in an unblinded standardized manner by two reviewers (APNJ and LMSM). Disagreements were resolved by consensus. The preferred reporting items for systematic reviews and meta-analyses (PRISMA) statement was used for guidance [7]. This study did not need ethical approval nor was individual patient consent needed as only data from published studies were used.

### Study selection

One of the criteria used to include studies in this review was whether they assessed the general SAPS III equation performance on predicting hospital mortality in an adult population (≥16 years-old) admitted to an ICU. Included studies needed to have evaluated at least both calibration and discrimination of the model. Calibration refers to whether the predicted probabilities of death in the hospital agree with the observed ones. Discrimination refers to the ability of the model to distinguish high-risk subjects from low-risk subjects. When reported in the included studies, calibration and discrimination data from customized SAPS III equations for major areas of the world were also reported here.

We decided to include only studies that used data collected after 2002, when data for original SAPS III were available [5,6]. This decision was made in order to avoid conclusions about performance of the model based on a different profile of treatments administered in a previous period of time.

### Quality assessment

Included studies were evaluated for quality according to the following items, based on a guideline for systematic review of prognostic studies [8]: 1) study participation: study population was clearly defined and described; 2) prognostic factor measurement: SAPS III was properly measured (that is, data were collected as described on the original study); 3) analysis: adequate description of the test methods for discrimination and calibration and sufficient presentation of data to assess adequacy of analysis.

### Data extraction

We developed a data extraction sheet. One author (APNJ) extracted the following data from included studies: number of patients, mean or median age, percentage of female patients and percentage of patients admitted for a surgical reason (defined as ICU admission from the operating room when not explicitly stated). The second author (LMSM) checked the extracted data. Authors of the included studies were contacted by email to complete the missing data that were required for characterizing the studies. When the authors could not be contacted, did not reply or their answer was still unclear, empty fields were marked ‘Not Reported (NR)’.

For each included study, we described the reported calibration and discrimination. Calibration is usually measured by the Hosmer-Lemeshow (H-L) goodness-of-fit *H*- or *C*-statistics. These statistics test for significant departures from perfect calibration when *P*-values are greater than 0.05. The *H*-statistic is based on fixed cut points on the predictions (for example, deciles of risk) whereas the *C*-statistic is based on equally sized groups, based on probability of death. Additionally, calibration may be evaluated by the Cox test of calibration. In this case, logistic regression is used to verify the agreement between predicted and observed risks.

In studies in which both the general and the customized SAPS III showed significant departure from perfect calibration, we assessed if authors performed a customization and if this new model was an adequate calibration for the study population. First-level customization is performed using logistic regression analysis by computing a new logistic coefficient while maintaining the same variables with the same weights as the original model [9].

Discrimination is assessed measuring the area under the receiver operator characteristic (aROC) curve. aROC and its 95% CI were calculated. Discrimination was considered excellent, very good, good, moderate or poor with aROC values of 0.9 to 0.99, 0.8 to 0.89, 0.7 to 0.79, 0.6 to 0.69 and ≤0.6, respectively. When available, we also presented the standardized mortality ratio (SMR) for hospital mortality reported in the studies. SMR is calculated by dividing observed hospital mortality by the predicted hospital mortality.

As classical calibration tests (such as H-L statistics) are extremely sensitive to sample size [10,11], we decided to evaluate if there was a correlation between sample size and calibration tests. Spearmann correlation coefficient was calculated between the number of included patients and the value of H-L statistics found. For studies presenting both the *H*- and *C*-statistic, we choose the one that reported the lower value. Analysis was performed on SPSS version 22.0 (SPSS Inc.).

## Results

### Study characteristics

**Figure 1:**
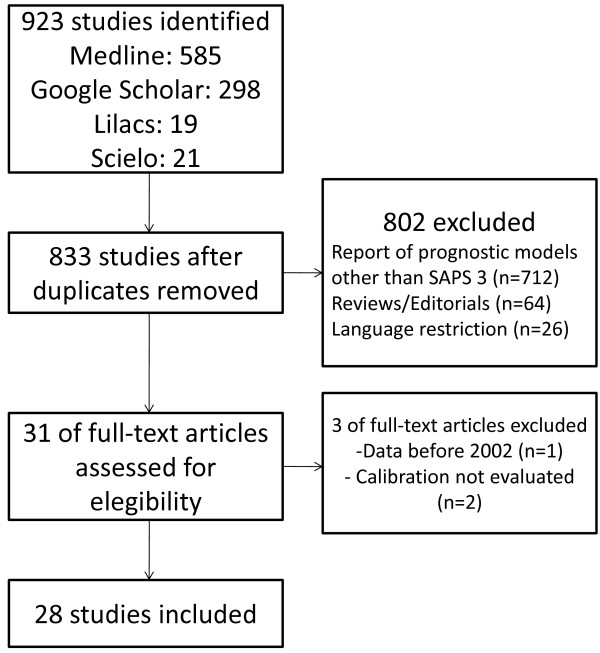
**Search strategy.** SAPS, simplified acute physiology score.

Out of 923 studies initially identified, 31 full-text articles were assessed for eligibility. Two were excluded for not performing any measure of calibration [12,13] and one for using data collected before 2002 [14]. Twenty-eight studies were included in this analysis (Figure 1).

Table 1 shows the characteristics of the included studies. According to major geographic regions defined in the original SAPS III study, there were ten studies (35.7%) performed in Central and South America [15-24], one (3.6%) in North America [25], eight (34.8%) in Australasia [26-33], three (10.7%) in Central and Western Europe [34-36], two (7.1%) in Northern Europe [37,38] and four (14.3%) in Southern Europe and Mediterranean countries [39-42]. Most studies (53.5%) were performed in a single ICU. Included studies examined the total number of 63,261 patients, ranging from 95 to 28,357.

**Table 1:** Study characteristics

**Study**	**Setting**	**Study period**	**Number of patients**	**Age (years)**	**Female (%)**	**Surgical admission (%)**	**Hospital mortality (%)**
Soares, 2006 [15]	1 ICU, Brazil	Jan 2003 to Dec 2005	952	Mean: 58.3	45.3	63.5	33.5
Serrato, 2007 [16]	1 ICU, Mexico	Jan 2006 to May 2006	95	Mean: 59	49.0	28.0	20.0
Ledoux, 2008 [34]	1 ICU, Belgium	Dec 2005 to Jul 2006	802	Median: 66	39.4	71.1	17.5
Duke, 2008 [26]	1 ICU, Australia	Oct 2005 to Dec 2007	1,741	Median: 66	47.4	39.4	11.3
Tsai, 2008 [31]	1 ICU, Taiwan	Jan 2002 to Dec 2006	104	Mean: 51	42.3	61.4	76
Sakr, 2008 [35]	1 ICU, Germany	Aug 2004 to Dec 2005	1,851	Mean: 61.6	36.6	85.8	9.0
Metnitz, 2009 [36]	22 ICU, Austria	Oct 2006 to Feb 2007	2,060	Mean: 64.9	42.0	57.5	21.7
Capuzzo, 2009 [39]	2 ICU, Italy	Jan 2006 to Sep 2007	684	Median: 73	37.0	81.8	19.6
Strand, 2009 [37]	2 ICU, Norway	Jun 2006 to Dec 2007	1,862	Median: 63	36.0	29.4	23.6
Alves, 2009 [17]	1 ICU, Brazil	Jan 2006 to Dec 2006	350	Mean: 73.4^a^	48.0	31.1	30.6
Mbongo, 2009 [40]	1 ICU, Spain	Jan 2006 to Dec 2006	864	Mean: 60.7	34.7	86.3	8.2
Poole, 2009 [41]	147 ICU, Italy	Feb 2007 to Dec 2007	28,357	Mean: 65.8	40.7	53.5	29.6
				Median: 70			
Maccariello, 2010 [18]	11 ICU, Brazil	Jan 2007 to Jul 2008	244	Mean: 69.5	43.0	19.0	68.0
Silva Junior, 2010 [19]	2 ICU, Brazil	Mar 2008 to Mar 2009	1,310	Mean: 67.1	60.5	100	10.8
Khwannimit, 2010 [27]	1 ICU, Thailand	Jan 2005 to Dec 2010	1,873	Median: 62	41.1	0	28.6
Soares, 2010 [20]	28 ICU, Brazil	Aug 2007 to Sep 2009	717	Mean: 61.2	51.0	64.0	30.0
Khwannimit, 2011 [28]	1 ICU, Thailand	Mar 2007 to Aug 2009	2,022	Median: 62	40.0	0	26.1
Lim, 2011 [32]	1 ICU, South Korea	Mar 2008 to Feb 2009	633	Mean: 60	37.0	0	31.0
Costa e Silva, 2011 [21]	6 ICU, Brazil	Nov 2003 to Jun 2005	366	Mean: 57.1	41.0	23.5	67.8
Christensen, 2011 [38]	1 ICU, Denmark	Jan 2007 to Dec 2007	469	NR	66.3	63.5	17.7
Juneja, 2012 [29]	1 ICU, India	Jul 2008 to Sep 2009	653	Mean: 58.5	42.1	0	15.8
Nassar Junior, 2012 [22]	3 ICU, Brazil	Jul 2008 to Dec 2009	5,780	Median: 66	52.7	20.9	9.1
Keegan, 2012 [25]	3 ICU, USA	Jan 2006 to Dec 2006	2,596	Mean: 63.2	45.2	19.8	10.9
Nassar Junior, 2013 [23]	3 ICU, Brazil	Jul 2008 to Dec 2009	1,015	Median: 61	40.4	0	2.1
De Oliveira, 2013 [24]	1 ICU, Brazil	May 2006 to Jan 2007	501	Mean: 46	34.5	100	7.8
Khwannimit, 2013 [30]	1 ICU, Thailand	Jan 2005 to Dec 2010	880	Median: 59	42.4	30.6	57.4
Lim, 2013 [33]	22 ICU, South Korea	Jul 2010 to Jan 2011	2,309^b^	Median: 62	35.3	39.8	20.1
López-Caler, 2013 [42]	6 ICU, Spain	Jan 2006 to Oct 2007	2,171	Mean: 61.4	NR	37.2	16.0

^a^Only patients aged ≥60 years were included; ^b^development cohort in which a customized SAPS III equation was developed. NR, not reported.

Although most studies aimed to validate SAPS III on a broad population in medical, surgical or mixed ICUs, nine studies (32.1%) applied SAPS III to patients with specific conditions such as cancer [15,20], elderly [17], acute kidney injury (AKI) [18,21,31], acute coronary syndromes [23], septic shock [30] and transplant patients [24].

Khwannimit *et al*. performed three studies in the same ICU and enrolled some patients in more than one study [27,28,30]. Lim *et al*. included 4,617 patients, but the SAPS III general equation was only assessed on 2,309 patients, a ‘development cohortʼ in which a customized Korean SAPS III was generated [33]. The Australasian SAPS III equation was assessed in the entire population, but we only extracted data from the development cohort for comparison with SAPS III general equation data on calibration and SMR.

### Study quality

Study quality assessment is shown in Table 2. Three studies did not calculate SAPS III with data collected within one hour from admission. Tsai *et al*. aimed to validate SAPS III at dialysis commencement in a population supported by extracorporeal membrane oxygenation [31]. Although they also calculated SAPS III at ICU admission, they only evaluated discrimination at this point. Maccariello *et al*. applied SAPS III in an AKI population at the start of renal replacement therapy [18]. Costa e Silva *et al*. also studied an AKI population and applied SAPS III on AKI diagnosis and on nephrology consultation day, using data collected during the previous 24 hours [21].

**Table 2:** Quality assessment of included studies

**Study**	**Study participation**	**Prognostic factor measurement**	**Analysis**
Soares, 2006 [15]	Yes	Yes	Yes
Serrato, 2007 [16]	Yes	Yes	Yes
Ledoux, 2008 [34]	Yes	Yes	Yes
Duke, 2008 [26]	Yes	Yes	Yes
Tsai, 2008 [31]	Yes	No	Yes
Sakr, 2008 [35]	Yes	Yes	Yes
Metnitz, 2009 [36]	Yes	Yes	Yes
Capuzzo, 2009 [39]	Yes	Yes	Yes
Strand, 2009 [37]	Yes	Yes	Yes
Alves, 2009 [17]	Yes	Yes	Yes
Mbongo, 2009 [40]	Yes	Yes	Yes
Poole, 2009 [41]	Yes	Yes	Yes
Maccariello, 2010 [18]	Yes	No	Yes
Silva Junior, 2010 [19]	Yes	Yes	Yes
Khwannimit, 2010 [27]	Yes	Yes	Yes
Soares, 2010 [20]	Yes	Yes	Yes
Khwannimit, 2011 [28]	Yes	Yes	Yes
Lim, 2011 [32]	Yes	Yes	Yes
Costa e Silva, 2011 [21]	Yes	No	Yes
Christensen, 2011 [38]	Yes	NR	Yes
Juneja, 2012 [29]	Yes	Yes	Yes
Nassar Junior, 2012 [22]	Yes	Yes	Yes
Keegan, 2012 [25]	Yes	Yes	Yes
Nassar Junior, 2013 [23]	Yes	Yes	Yes
De Oliveira, 2013 [24]	Yes	Yes	Yes
Khwannimit, 2013 [30]	Yes	Yes	Yes
Lim, 2013 [33]	Yes	Yes	Yes
López-Caler, 2013 [42]	No	Yes	Yes

NR, not reported.

### Calibration and discrimination

Only one study opted for the Cox calibration test instead of H-L statistics to assess calibration [41]. A non-significant departure from the perfect calibration was found in eleven studies (42.8%) (Table 3) [15,16,18,19,21,29,31,32,37,38,40].

**Table 3:** Performance of general and customized SAPS III equations for major geographic regions

**Study**	**Calibration for general SAPS III**	**Calibration for customized SAPS III**	**aROC (95% CI)**	**SMR (95% CI) for general SAPS III**	**SMR (95% CI) for customized SAPS III**
**Central and South America**					
Soares, 2006 [15]	*C*: 13.637 (*P* = 0.092)	*C*: 9.132 (*P* = 0.331)	0.87 (0.85 to 0.90)	1.19 (1.04 to 1.37)	0.95 (0.84 to 1.07)
Serrato, 2007 [16]	*C*: 6.64 (*P* >0.1)	NR	0.86 (0.825 to 0.895)	0.81	NR
Alves, 2009 [17]	*H*: 16.42 (*P* = 0.037)	*H*: 16.66 (*P* = 0.034)	0.881 (0.843 to 0.913)	1.10 (0.90 to 1.33)	0.86 (0.70 to 1.04)
	*C*: 17.57 (*P* = 0.025)	*C*: 15.95 (*P* = 0.047)			
Maccariello, 2010 [18]	*C*: 10.16 (*P* = 0.254)	*C*: 9.33 (*P* = 0.315)	0.82 (0.76 to 0.88)	1.26 (1.10 to 1.46)	1.04 (0.92 to 1.18)
Silva Junior, 2010 [19]	*C*: 10.47 (*P* = 0.234)	NR	0.86 (0.83 to 0.88)	1.04 (1.03 to 1.07)	NR
Soares, 2010 [20]	*C*: 15.804 (*P* = 0.045)	*C*: 12.607 (*P* = 0.126)	0.84 (0.81 to 0.87)	1.29 (1.09 to 1.53)	1.02 (0.87 to 1.19)
Costa e Silva, 2011 [21]	*H* (DD): 6.86 (*P* = 0.551)	*H* (DD): 6.33 (*P* = 0.610)	DD: 0.73 (0.67 to 0.78)	DD: 1.35 (1.07 to 1.63)	DD: 1.09 (0.83 to 1.35)
	*H* (NCD): 10.47 (*P* = 0.163)	*H* (NCD): 13.22 (*P* = 0.113)	NCD: 0.80 (0.73 to 0.86)	NCD: 1.15 (0.75 to 1.55)	NCD: 1.00 (0.61 to 1.39)
Nassar Junior, 2012 [22]	*C*: 226.6 (*P* <0.001)	NR	0.855 (0.846 to 0.864)	0.46 (0.37 to 0.54)	NR
Nassar Junior, 2013 [23]	*C*: 51.8 (*P* <0.001)	NR	0.804 (0.779 to 0.828)	0.31 (0.11 to 0.50)	NR
De Oliveira, 2013 [24]	*H*: 59.41 (*P* <0.001)	*C*: 123.49 (*P* <0.001)	0.696 (0.607 to 0.786)	1.94 (1.38 to 2.64)	1.88 (1.34 to 2.56)
	*C*: 155.57 (*P* <0.001)	*H*: 45.6 (*P* <0.001)			
**North America**					
Keegan, 2012 [25]	*C*: 36.6 (*P* <0.05)	NR	0.801 (0.785 to 0.816)	0.66 (0.69 to 0.75)	NR
**Australasia**					
Duke, 2008 [26]	*H*: 36.15 (*P* = 0.009)	*H*: 27.37 (*P* = 0,06)	0.88 (0.85 to 0.90)	0.84 (0.724 to 0.969)	0.92 (0.80 to 1.06)
	*C*: ? (*P* = 0.019)				
Tsai, 2008 [31]	*H*: 5.445 (*P* = 0.71)	NR	0.73	NR	NR
Khwannimit, 2010 [27]	*H*: 106.7 (*P* <0.001)	*H*: 98.2 (*P* <0.001)	0.933 (0.921 to 0.944)	0.86 (0.79 to 0.93)	0.92 (0.85 to 0.99)
	C: 101.2 (*P* <0.001)	*C*: 96.2 (*P* <0.001)			
Khwannimit, 2011 [28]	*H*: 101.6 (*P* <0.001)	*H*: 79.9 (*P* <0.001)	0.916 (0.902 to 0.929)	0.81 (0.74 to 0.88)	0.88 (0.80 to 0.96)
	*C*: 176.3 (*P* <0.001)	*C*: 170 (*P* <0.001)			
Lim, 2011 [32]	*C*: 3.174 (*P* = 0.923)	*C*: 3.286 (*P* = 0.915)	0.78 (0.75 to 0.81)	0.72 (0.62 to 0.83)	0.78 (0.67 to 0.89)
Juneja, 2012 [29]	HL(?): 13.12 (*P* = 0.108)	NR	0.901 (0.871 to 0.932)	0.763 (0.628 to 0.918)	NR
Khwannimit, 2013 [30]	*H*: 39.4 (*P* <0.001)	NR	0.817 (0.790 to 0.845)	0.97 (0.89 to 1.06)	NR
	*C*: 49.6 (*P* <0.001)				
Lim, 2013 [33]	*H*: 123.06 (*P* <0.001)	*H*: 73.53 (*P* <0.001)	0.829 (0.82 to 0.86)	0.72 (0.65 to 0.78)	0.78 (0.71 to 0.85)
	*C*: 118.45 (*P* <0.001)	*C*: 70.52 (*P* <0.001)			
**Central and Western Europe**					
Ledoux, 2008 [34]	*C*: 16.59 (*P* = 0.035)	*C*: 8.30 (*P* = 0.405)	0.85 (0.82 to 0.89)	0.82 (0.70 to 0.93)	0.96 (0.84 to 1.08)
Sakr, 2008 [35]	*H*: 211.84 (*P* <0.001)	*H*: 177.37 (*P* <0.001)	0.84 (0.81 to 0.88)	NR	NR
	*C*: 208.49 (*P* <0.001)	*C*: 126.79 (*P* <0.001)			
Metnitz, 2009 [36]	*H*: 100.18 (*P* <0.001)	*H*: 51.56 (*P* <0.001)	0.82	0.79 (0.74 to 0.85)	0.86 (0.80 to 0.92)
	*C*: 90.29 (*P* <0.001)	*C*: 45.61 (*P* <0.001)			
**Northern Europe**					
Strand, 2009 [37]	*C*: 17.40 (*P* = 0.066)	*C*: 18.25 (*P* = 0.051)	0.81 (0.79 to 0.93)	0.71 (0.65 to 0.78)	0.74 (0.68 to 0.81)
Christensen, 2011 [38]	HL(?): 9.23 (*P* = 0.51)	NR	0.69 (0.63 to 0.75)	NR	NR
**Southern Europe and Mediterranean countries**					
Capuzzo, 2009 [39]	*H*: 23.36 (*P* = 0.002)	*H*: 25.73 (*P* = 0.001)	0.835 (0.794 to 0.876)	0.83 (0.77 to 0.89)	0.81 (0.75 to 0.87)
	*C*: 22.47 (*P* = 0.004)	*C*: 26.19 (*P* = 0.001)			
Mbongo, 2009 [40]	*C*: 8.57 (*P* = 0.38)	*C*: 7.5 (*P* = 0.48)	0.917 (0.880 to 0.954)	0.71 (0.56 to 0.90)	0.69 (0.55 to 0.87)
Poole, 2009 [41]	U^a^: 2,035,9 (*P* <0.001)	U^a^: 1,929.2 (*P* <0.001)	0.855 (0.851 to 0.860)	0.73 (0.72 to 0.75)	0.73 (0.72 to 0.75)
López-Caler, 2013 [42]	HL(?): 20.05 (*P* <0.05)	0.855 (0.851 to 0.860)	0.845 (0.821 to 0.869)	0.89 (0.80 to 0.98)	0.86 (0.77 to 0.95)

aROC, area under the receiver operator characteristic curve; SAP, simplified acute physiology score; SMR, standardized mortality ratios; *H*, Hosmer-Lemeshow *H*-statistic; *C*, Hosmer-Lemeshow *C*-statistic; HL(?), Hosmer-Lemeshow statistic unspecified; NR, not reported; DD, acute kidney injury diagnosis-day; NCD, day of nephrology consultation. ^a^U statistics quantifies the difference from the null hypothesis, that is, a good fit of the model in the studied population when Cox calibration test is used.

A positive correlation was found between sample size and the value of H-L statistics in the 27 studies that used it to assess calibration (*r* = 0.747; *P* <0.001), meaning that larger studies correlated with higher H-L values representing greater power to detect mis-calibration.

Nineteen studies (67.9%) also assessed calibration for the customized SAPS III equation for their major geographic region (Table 3) [15,17,18,20,21,24,26,27,32-37,39-42]. A non-significant departure from perfect calibration was found in six studies, in which the general equation also did not show a statistically significant mis-calibration [15,18,21,32,37,40]. Three studies [20,26,34], did not show a significant departure from perfect calibration for SAPS III customized equations, unlike the general SAPS III equation, which was statistically significantly mis-calibrated in those studies. In all remaining 10 studies, significant departures from perfect calibration were found for both customized and general equations [17,24,27,28,32,33,35,36,39,41,42].

Five studies (17.9%), in which both general SAPS III equation and major geographic customized equations were statistically significantly different from perfect calibration, performed a regional customization. All studies developed a model which then did not show a statistically significant departure from perfect calibration for their populations [27,28,33,35,36]. Only two of these studies were multicenter [33,36].

Discrimination was always very good (aROC 0.80 to 0.89) or excellent (aROC ≥0.90) in 24 studies (85.7%) [15-23,25-30,33-37,39-42]. None of the studies showed poor discrimination (Table 3).

### Mortality prediction

Twenty-five studies (89.3%) reported SMR for hospital mortality [15-30,32-34,36,37,39-42]. The SAPS III general equation underestimated hospital mortality in six studies (24%) [15,18-21,24] and overestimated it in 15 (60%) [22,23,25-29,33,34,36,37,39-42]. Two studies (8%) reported an SMR lower than 1 but did not report the 95% CI [16,32] (Table 3).

Out of the 19 studies that assessed the customized SAPS III equation for their major geographic region, the customized SAPS III still overestimated hospital mortality in 10 studies (52.6%) [27,28,32-34,36,37,39-41]. In one study, the customized SAPS III also underestimated hospital mortality [24]. However, in six studies, switching from the general to the customized equation was associated with better mortality estimation [15,18,20,21,26,34] (Table 3).

## Discussion

This systematic review of literature identified 28 studies that addressed SAPS III performance in external populations. As SAPS III enrolled patients from diverse countries, it would be reasonable to suppose it would perform well in external validation studies. However, this was not the rule. SAPS III general equations discriminated very well in almost all studies, but calibration tests suggested significant departures from perfect calibration in most of them, especially in large studies (as should be expected by the extensive use of the H-L, *C*- and *H*-statistics). SAPS III customized equations have not delivered a better performance than the general equation in most of the studies. However, local customization has provided non-statistically significant departures from perfect calibration for SAPS III in all studies that performed it.

Calibration refers to the agreement between observed and predicted risks. A non-statistically significant departure from perfect calibration, that is, a *P*-value higher than 0.05, in an external validation study means the prognostic model predicts mortality risk adequately in this population. Therefore, this population and the one in which the original model was developed are similar. A series of reasons can be responsible for significant departures from perfect calibration. Among these are sampling bias, variations in case-mix and temporal bias (either in the process of care or in the case-mix).

SAPS III original cohort included patients with a median age of 64 years; 39.4% of the patients were female and 51.5% of them were surgical (elective and emergency). Differences in case-mix were important in the included studies. Mean or median age varied from 46.0 to 73.4 years-old; female patients varied from 34.5 to 66.3%; and the proportion of surgical patients varied from 0 to 100%. Interestingly, if we choose two studies with similar populations according to these three variables - studies performed by Metnitz [36] and by Strand [37] - we observe that a significant departure from perfect calibration was found in the former, but not in the latter. Even more interestingly, SAPS III had non-significant departures from perfect calibration in studies that included higher proportions of surgical patients [15,19,20,34] than the original SAPS III study, but that also happened in a study that included only medical patients [29]. Of course, not only these three variables explain similar or different case-mixes. Poor SAPS III performance may also be due to differences in healthcare provisions [43] and end-of-life policies [44], for example.

Another point that deserves attention is the application of a prognostic model in a population admitted to ICU because of a specific diagnosis. SAPS III had a non-significant departure from perfect calibration and still had very good discrimination in a common ICU syndrome - AKI - when calculated with data collected on the diagnosis day, on the day of nephrology consultation [21] or at the start of renal replacement therapy [18,31]. On the other hand, SAPS III was statistically significantly mis-calibrated in an assessment of septic shock patients, although it discriminated very well [30]. Two studies that included patients with a specific diagnosis at admission, however, showed very poor performance of SAPS III. In transplant patients, SAPS III was statistically significantly mis-calibrated, had only moderate discrimination and underestimated hospital mortality [24]. In acute coronary syndrome patients, SAPS III also was statistically significantly mis-calibrated and overestimated hospital mortality, although it discriminated well in this population [23]. These findings are not unexpected. General prognostic models usually do not perform well in specific subgroups of patients because they may be under-represented in the developed cohort [6]. For some specific diagnoses, a specific prognostic model may be an attractive alternative [23,45].

Temporal bias is another problem frequently reported as a reason for significant differences from perfect calibration. SAPS III was developed with 2002 data. It is possible that changes in case-mix or clinical practice over time may limit the application of a prognostic model. Analyzing study periods in Table 1 and calibrations in Table 3, it is not possible to conclude that studies that collected data in a specific period of time also found a better SAPS III performance.

A large database of patients from seven different major geographic regions allowed SAPS III customized equations for each region. In the original study, these customized equations were not statistically significantly different from perfect calibration [6]. Among included studies that also assessed customized equations, there were only three in which the customized equation did not show a statistically significant mis-calibration in a population in which the general equation also showed the same findings. Despite being developed to fit better, these equations may not be representative of populations from a specific region. For example, SAPS III included 1,756 Australasian patients, but these patients were only from Australia, Hong Kong and India. Khwannimit *et al*. performed their studies in a Thai population, and the Australasian SAPS III was also statistically mis-calibrated [27,28,30]. Similar findings were identified by Lim *et al*. in a broad South Korean population [33].

A better explanation for SAPS III departures from perfect calibration may be the limitations of the statistics methods used to evaluate performance. Calibration statistics (specially H-L goodness-of-fit statistics) present several problems, mainly the fact that they are very sensitive to larger sample sizes because larger studies have more power to detect departures from perfect calibration [10,46]. Thus, even small deviations from the perfect calibration in larger studies may be associated with a *P*-value >0.05. An interesting study compared the performance of SAPS II, APACHE II and the mortality predict model II (MPM-II) in a Dutch population of 42,139 patients. All models showed statistically significant departures from perfect calibration, both on the H-L and on the Cox calibration test. However, when prognostic models were assessed in subsamples drawn from the database, performance was better. There was a tendency to reject the model when these samples increased [11]. Our review suggested a correlation between larger studies and higher values in H-L statistics, as we should expect.

Although neither the SAPS III general equation nor its customized equations for major geographic areas did not show statistical goodness-of-fit in most studies, all studies that performed a customization, developed local models that did not show significant departures from perfect calibration and had a very good discrimination. The development of country-specific equations was previewed in the original SAPS III description [6,36]. These findings highlight the value and weight of variables included in SAPS III, as first-line customizations provided valid models in all studies. Models with only a few parameters, such as SAPS III, are quite stable and can easily be turned into a statistically calibrated model with a first-level customization [47]. However, only two studies that developed local models were multicenter. Thus, one may argue against considering that a model is valid for a country or region if it was customized only with data from a single ICU.

Discrimination refers to how well the model discriminates between an individual who will live and one who will die. Good, very good or excellent discrimination was found in almost all studies. Only two studies had an aROC <0.7 [24,38]. Both were single center and one of them was performed in transplant patients [24], a very specific population. We believe this is a reassuring finding for those using SAPS III for clinical or research purposes in general populations of critically ill patients.

In addition to limitations of the included studies, one possible limitation of this review is how databases were searched. Although we did not restrict our search to English language articles, there were language restrictions that may have caused us to miss some studies. Also, we performed searches on databases that include only studies from Latin America (Lilacs), and from Latin America, Portugal, Spain and South Africa (Scielo). That may have been responsible for the high number of Latin American and Spanish studies analyzed, which encompassed almost half of the included studies. However, all studies, except for two [16,17], were also indexed on Medline. One of them was retrieved from Scielo [17] and the other from Google Scholar [16].

## Conclusions

Although SAPS III was the first intensive care prognostic model developed with patient data from different regions of the world, its performance in external validation studies was far from perfect. Even its major geographic customized equations showed significant departures from perfect calibration. Local and country customizations, on the other hand, improved its performance. Discrimination was almost always very good or excellent. We believe that SAPS III is a reliable, simple and easy prognostic model to be used in clinical practice, but it should be customized before routine application in local settings. This statement is possibly (probably) true for all general outcome prediction models. In addition to that, it seems that SAPS III should not be used to assess patients admitted with specific diagnoses.

## Key messages

● SAPS III showed significant departure from perfect calibration in most studies

● There was a positive correlation between larger samples and higher H-L values for SAPS III. As calibration statistics are very sensitive to sample sizes, this is probably the best explanation for the significant departures from perfect calibration found in the larger studies

● SAPS III discrimination is very good

● First-level customization improved SAPS III performance in all studies in which it was accomplished, although most of them were single-center studies

● SAPS III is a reliable prognostic model to be used in clinical practice, but it should be customized before routine application in local settings.

## Abbreviations

AKI: acute kidney injury; aROC: area under the receiver operator characteristic curve; H-L: Hosmer-Lemeshow; PRISMA: preferred reporting items for systematic reviews and meta-analyses; SAPS: simplified acute physiology score; SMR: standardized mortality ratio.

## Competing interests

The authors declare that they have no competing interests.

## Authors’ contributions

APNJ conceived of this study, carried out the search queries, reviewed the articles, assessed their quality, extracted the data and drafted the manuscript. LMSM carried out the search queries, reviewed the articles, extracted the data and helped to draft the manuscript. RM participated in the design and coordination of the study and helped to draft the manuscript. All authors read and approved the manuscript.
